# The Relationship of *APOE* ε4, Race, and Sex on the Age of Onset and Risk of Dementia

**DOI:** 10.3389/fneur.2021.735036

**Published:** 2021-10-20

**Authors:** Danielle S. Powell, Pei-Lun Kuo, Riaz Qureshi, Sally B. Coburn, David S. Knopman, Priya Palta, Rebecca Gottesman, Michael Griswold, Marilyn Albert, Jennifer A. Deal, Alden L. Gross

**Affiliations:** ^1^Department of Epidemiology, Johns Hopkins University, Baltimore, MD, United States; ^2^Center on Aging and Health, Johns Hopkins University, Baltimore, MD, United States; ^3^Department of Neurology, Mayo Clinic, Rochester, MN, United States; ^4^Division of General Medicine, Columbia University Medical Center, New York, NY, United States; ^5^Department of Neurology, Johns Hopkins University, Baltimore, MD, United States; ^6^Biostatistics, School of Medicine, University of Mississippi Medical Center, Jackson, MS, United States

**Keywords:** apolipoprotein ε allele, dementia, genetics, race, sex, cognitive aging, epidemiology

## Abstract

**Objective:** To investigate whether *APOE* ε4 genotype—an established risk factor for dementia—is associated with earlier age at diagnosis in addition to increased risk overall and in secondary analysis by race and sex.

**Methods:** We followed up 13,782 dementia-free individuals (*n* = 10,137 White, *n* = 3,645 Black, baseline age 60–66 years) in the Atherosclerosis Risk in Communities study for up to 30 years. Dementia was operationalized using standardized algorithms incorporating longitudinal cognitive change, proxy report, and hospital or death certificate dementia codes. We used a mixture of generalized gamma distributions to simultaneously estimate time to dementia, time to dementia-free death, and the proportion of individuals with dementia, by *APOE* ε4 status (≥1 vs. no alleles).

**Results:** Median age of dementia onset among *APOE* ε4 carriers was 81.7 (Blacks) and 83.3 years (Whites) compared with 82.6 (Blacks) and 85.7 years (Whites) in non-*APOE* ε4 carriers (*p* > 0.05 Blacks; *p* < 0.01 Whites). Age of dementia diagnosis did not differ by sex in ε4 carriers, but among non-carriers, average age was earlier in males than females regardless of race. *APOE* ε4 carriers had on average a higher proportion of diagnoses; results did not differ by race or sex.

**Conclusions:** *APOE* ε4 carrier status is associated with earlier age of dementia diagnosis with differences across race and sex. These findings clarify the causal role of *APOE* in dementia etiology, which could help better identify at-risk subgroups and may help facilitate better research trial recruitment and design.

## Introduction

Carriers of the apolipoprotein (*APOE*) ε4 allele contribute to 7% of incident global dementia cases and have double the risk of developing mild cognitive impairment (MCI) and dementia (1, 2) than have non-carriers. Risk conferred by *APOE* ε4 varies by race and sex (3–6). However, most prior studies have been conducted in clinical, rather than population-based, samples. Although risk of dementia is greater in Blacks compared with Whites, the relative effect of *APOE* ε4 may be greater in Whites compared with Blacks, potentially because a larger component of the risk seen in Blacks is due to other health factors such as vascular disease (7–9).

Additionally, most prior studies of *APOE* ε4 and dementia only estimate risk of dementia. By contrast, time to dementia by age (10, 11) allows for expansion upon current knowledge of the appropriate design and timing of dementia trials and may improve identification of people at risk for dementia at more appropriate ages. An additional limitation of prior studies is that most have failed to account for death, which can bias risk estimates for dementia (12, 13). One of the few studies to do so found over two times greater risk for Alzheimer's disease among White individuals who are heterozygous ε4 carriers as compared with non-carriers (11).

In this study, we comprehensively evaluated *APOE* ε4's association with dementia by race and sex (14, 15). We jointly modeled both age of diagnosis and risk of dementia diagnosis or death, accounting for the competing risk of death (13) over 30 years in the Atherosclerosis Risk in Communities (ARIC) study. Investigation of both age of dementia diagnosis and risk for diagnosis has not been comprehensively evaluated within the same cohort or has not considered the competing risk of death in analysis by using a large longitudinal sample of older adults, additionally evaluating potential differences by race and sex. The use of the modeling technique provided allows for direct estimates of the both time to dementia diagnosis and risk of diagnosis within the same model and allows for greater characterization of APOE ε4 allele carrier status on risk of dementia than isolated models may provide. We hypothesized that the presence of any ε4 alleles is associated with earlier age of dementia diagnosis and greater risk of diagnosis in females and/or White participants.

## Methods

### The Atherosclerosis Risk in Communities Study

**Figure 1:**
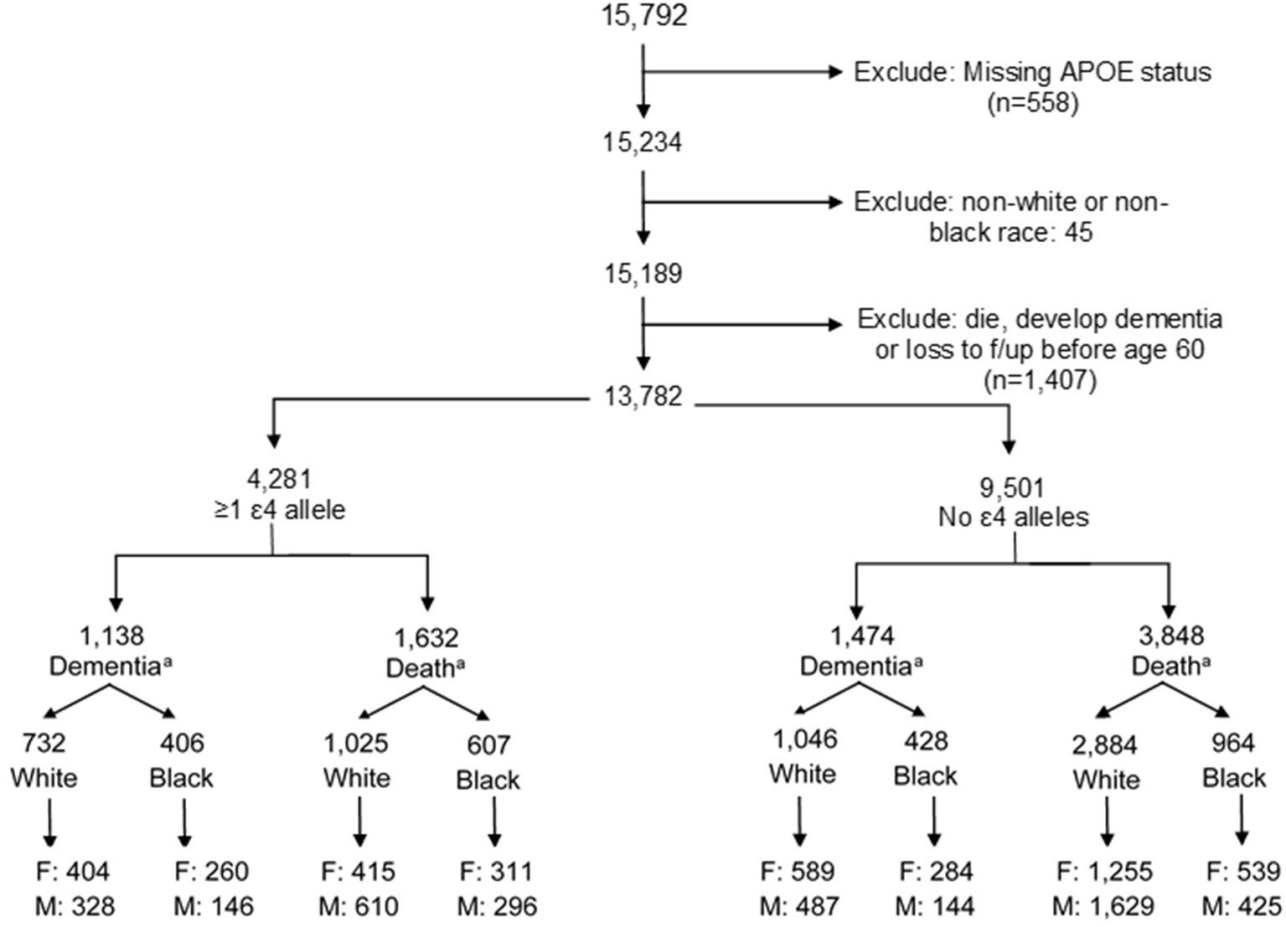
Creation of analytic study sample. F, female; M, male. ^a^Final dementia or death numbers by ε4 status and stratification by males and females indicate the number of participants who experienced a dementia diagnosis or dementia-free death. Numbers do not add up to the total by ε4 allele status due to those participants who were censored at the end of study follow-up alive and without dementia.

ARIC is a prospective longitudinal cohort of 15,792 community-dwelling participants aged 45–64 years when recruited in 1987–1989 from four US communities (Forsyth County, NC; Jackson, MS; Minneapolis, MN; and Washington County, MD). The present study uses data from visits between 1987 and 2017 (visit 1: 1987–1989; visit 2: 1991–1992; visit 3: 1994–1995; visit 4: 1997–1998; visit 5: 2011–2013; and visit 6: 2016–2017). Our analysis includes both Black and White participants, a strength of the ARIC cohort. Exclusion criteria included the following: missing *APOE* status (*n* = 558), non-White or non-Black participants due to small sample size (*n* = 45), or diagnosis of dementia or death prior to age 60 (*n* = 1,407) to remove potential cases of early-onset dementia. Our final analytic sample included 13,782 participants (Figure 1; *n* = 10,137 White, *n* = 3,645 Black).

### Exposure Measurement: *APOE* Status

Genotyping of the *APOE* ε4 allele was completed using TaqMan system (Applied Biosystems, Foster City, CA, USA). A plurality of participants was ε3/ε3 (58.8% Whites and 45.0% Blacks). Few participants were homozygous for ε4 or ε2 (ε4/ε4: 2.1% Whites, 4.5% Blacks; ε2/ε2: 0.8% White, 1.4% Blacks). Due to the small number of ε4/ε4 participants (*n* = 375), particularly for stratified analyses, *APOE* status was dichotomized as “ε4 carrier” (≥1 ε4 alleles, *N* = 4,281) compared with “no ε4 alleles” (*N* = 9,501).

### Outcome Ascertainment: Dementia

Dementia diagnosis was ascertained for all participants, including those who did not return for clinic visits or died during follow-up. Dementia was defined *via* guidelines from National Institute on Aging-Alzheimer's Association (NIA-AA) workgroups and *Diagnostic and Statistical Manual of Mental Disorders*, 5th edition (DSM-5). Diagnosis was determined *via* standardized algorithms, followed by review by expert panel composed of four physicians and four neuropsychologists as has been described in detail in prior analysis (16, 17). A full neuropsychological battery of 10 tests was administered at both visits 5 and 6 and a shorter neurocognitive battery at visits 2 and 4. All test batteries assessed the domains of memory, language, and executive function. The reader is referred to Knopman et al. (18) for additional information on ARIC protocols for dementia ascertainment. For participants who returned for either or both of these visits, dementia diagnosis was determined using longitudinal cognitive evaluations from prior visits. For those alive but who did not return, suspect dementia was identified when possible based on telephone cognitive assessment using the Telephone Interview for Cognitive Status Modified (TICSm) or proxy report (2011–2013), by Six-Item Screener (SIS) and AD8 or proxy report (2016–2017). The primary outcome of interest for our analysis includes the prior methods as well as additional dementia cases identified by surveillance for diagnosis *via* hospital discharge records or death certificate codes throughout follow-up (16, 19).

Date of dementia diagnosis or censoring was ascertained for all participants, allowing for near-continuous monitoring of diagnosis (16). Dementia diagnosis date was recorded as the date of the study visit or interview in which cognitive testing supported a dementia diagnosis, or as 6 months prior to evidence of diagnosis if provided *via* proxy, hospital, or death certificate codes to account for an expected lag in dementia ascertainment from these sources (17, 18).

### Death

Date of death is obtained in ARIC for every participant *via* the National Death Index, hospital records, or family reports.

### Covariates

Education and sex were recorded at study entry and were both self-reported. Education (measured in 1987–1989) was recorded as the highest grade or year completed and was categorized for analysis as “less than high school,” “high school or equivalent,” or “greater than high school.” Sex was recorded as male or female. Race was operationalized as Black vs. White for our analysis based on self-report racial identification by participants at baseline. The small number of participants who did not identify as Black or White race (*n* = 48) was excluded from the analysis.

### Statistical Analysis

To describe demographic characteristics and assess their distribution by *APOE* ε4 status by race and sex, we tested for differences using chi-square for categorical variables and *t*-tests for continuous variables after tabulating proportions and calculating means and standard deviations (SDs).

To test our hypotheses, we conducted survival analyses (20) to characterize both the timing and frequency of dementia or dementia-free death using age as the time metric and 60 years of age as the time origin. We used age as the time metric because it offers a more meaningful clinical interpretation than time-on-study and facilitates more careful adjustment for age (21). We treated participants older than 60 years at study entry as late entries (22). Participants started contributing time on study as early as 60 years and stopped contributing time at (1) a diagnosis of dementia, (2) dementia-free death, or (3) end of study follow-up (December 31, 2017) for participants who survived without dementia at the end of visit 6 (18). For those participants who dropped out, and thus did not receive a dementia diagnosis by all ascertainment methods, or who died dementia-free, the censoring date used was the same for all participants who remained alive and dementia-free at the end of follow-up. We chose this date to be beyond the time an individual would normally have been expected to have either died or received a dementia diagnosis. For our analysis, we selected 105 years (sensitivity analysis showed no difference in estimate with an earlier age selection of 95 years). The reader may reference Checkley et al. (13) for further discussion.

To assess risk of and time to dementia diagnosis by *APOE* status, the survival analyses used a mixture model of saturated generalized gamma distributions (13). This mixture model of parametric survival distributions uses maximum likelihood estimation to fit a competing risk model in which competing events are two mutually exclusive event outcomes. The model summarizes the relative time to event for each type of event as indicated by age of onset of diagnosis (dementia and dementia-free death), compares how *APOE* status modifies the time to dementia and thereby age of diagnosis, and compares how *APOE* status modifies the proportion of those experiencing dementia before death (13). Prior work, including in this cohort, suggests a significant *APOE* ε*4* by race interaction, suggesting a stronger effect and higher incidence of dementia in Whites than in Blacks (11, 14, 16). We investigate this interaction and that of reported differences by sex by inclusion of an interaction term in the model, as well as modeling all analyses were stratified by race, with further stratification by sex within each race.

We accounted for dementia-free death by considering it as a competing risk for dementia. Inclusion of dementia-free death as a competing risk acknowledges that individuals may die from another cause without having the opportunity to develop dementia. Thus, dementia-free death removes an individual from the ability to be observed for a diagnosis of dementia and may lead to the overestimation of absolute risk (12) if not accounted for. In our competing risk analysis, death is considered an outcome but is not the primary outcome of interest (dementia diagnosis). If an individual dies before a diagnosis of dementia, we have no way of knowing if they would have developed dementia had they not died. In this way, death is a competing risk for observing dementia. The overall risk of dementia by *APOE* status is therefore dependent upon the survival function for both dementia and for death. Readers who wish to learn more about competing risks and statistical analysis may see Andersen et al. (12).

### Sensitivity Analysis

A total of 136 participants in our analytic sample had an ε2/ε2 genotype. Although a small proportion of our population (2.2%), this genotype has a known protective effect for dementia, and so inclusion of this group in our reference group could potentially result in an underestimate of the association with ε4 and dementia. To address this concern, we conducted a sensitivity analysis excluding this group.

To confirm adequate model fit for the generalized gamma, results were compared with those of a Weibull non-parametric model. Final models were selected based on Akaike information criteria (AICs). Analyses were conducted using Stata version 14 (StataCorp. 2015. Stata Statistical Software: Release 14; StataCorp LP, College Station, TX, USA).

## Results

### Demographic and Clinical Characteristics

Of *N* = 13,782 participants, *n* = 4,281 (31.1%) had ≥1 *APOE* ε4 allele, *n* = 2,612 (18.9%) participants had an incident dementia diagnosis, and *n* = 5,480 (39.8%) died without dementia during the 30 years of follow-up. The median duration of follow-up was 16.7 years (SD: 7.3 years). Average age of entry into analysis was 62.5 years (SD: 1.5 years). The average age of reaching a study endpoint due to dementia diagnosis was 80.3 years (SD 6.5 years) or due to dementia-free death was 74.8 years (SD 7.5 years). Black participants overall had an earlier age of dementia diagnosis or ascertainment and an earlier age of dementia-free death than had White participants, regardless of *APOE* status. White *APOE* ε4 carriers had a similar age of dementia diagnosis of 83.3 and 83.4 years for males and females, respectively, yet demonstrated differing ages for non-ε4 carriers. Both Black and White females had a later age of dementia diagnosis in non-ε4 carriers than had males. Demographic characteristics are in Table 1: 73.6% were White, 45% were male, 40% had a high school education and some college, and 36% had a college degree or greater. General demographic characteristics did not differ by *APOE* ε4 status for our primary outcome of interest of dementia diagnosis. Participants who were dementia-free at death were more likely to be male (46% compared with 59% of those with a dementia diagnosis) and were less likely to be an *APOE* ε4 carrier than were those who experienced a dementia diagnosis. A significant interaction by ε4 status was noted across race and sex for age of dementia diagnosis (sex: *p* = 0.36 carriers, *p* = 0.001 non-carriers; race: *p* = 0.000 both) but not for proportion experiencing a dementia diagnosis (sex: *p* = 0.804; race: *p* = 0.299).

**Table 1:** Demographic characteristics of study population.

		**≥1** ***APOE*** **ε4 allele**				**No** ***APOE*** **ε4 allele**			
	**Overall**	**White**		**Black**		**White**		**Black**	
		**Male**	**Female**	**Male**	**Female**	**Male**	**Female**	**Male**	**Female**
No (%)	13,782	1,370 (9.9)	1,458 (10.6)	583 (4.2)	870 (6.3)	3,475 (25.2)	3,834 (27.8)	794 (5.8)	1,398 (10.1)
Age at study entry, mean (SD)	62.5 (1.5)	62.6 (1.5)	62.4 (1.5)	62.6 (1.5)	62.5 (1.4)	62.5 (1.5)	62.5 (1.4)	62.7 (1.6)	62.6 (1.5)
Education level, *n* (%)									
Less than HS	3,307 (24.0)	238 (17.4)	221 (15.2)	271 (46.5)	365 (42.1)	647 (18.7)	663 (17.3)	345 (43.7)	557 (39.9)
HS and some college	5,532 (40.2)	526 (38.4)	736 (50.5)	135 (23.2)	248 (28.6)	1,342 (38.7)	1,923 (50.2)	206 (26.11)	416 (29.8)
College and beyond	4,921 (35.8)	606 (44.2)	501 (34.4)	177 (30.4)	255 (29.4)	1,479 (42.7)	1,244 (32.5)	238 (30.2)	421 (30.2)
Dementia diagnosis, *n* (%)	2,612 (18.9)	328 (23.9)	404 (27.7)	146 (25.0)	260 (29.9)	457 (13.2)	589 (15.4)	144 (18.1)	284 (20.3)
Death prior to dementia, *n* (%)	5,480 (39.76)	610 (44.5)	415 (28.5)	296 (50.8)	311 (35.8)	1,629 (46.9)	1,255 (32.7)	425 (53.3)	539 (38.6)
Length of follow-up, mean (SD)	16.7 (7.3)	16.9 (7.2)	17.2 (7.0)	14.8 (7.1)	15.7 (7.1)	16.9 (7.4)	17.3 (7.4)	14.8 (7.2)	15.7 (7.3)
Age at dementia, mean (SD)	80.3 (6.5)	80.3 (6.3)	80.4 (5.9)	78.4 (6.3)	78.9 (6.4)	80.8 (6.8)	82.1 (6.4)	78.3 (6.3)	79.2 (6.4)
Age at dementia-free death, mean (SD)	74.8 (7.5)	76.8 (7.7)	78.5 (7.6)	74.1 (7.5)	75.3 (7.7)	76.7 (7.8)	77.6 (8.0)	73.9 (7.5)	75.0 (7.9)

### Age at Dementia Diagnosis

**Figure 2:**
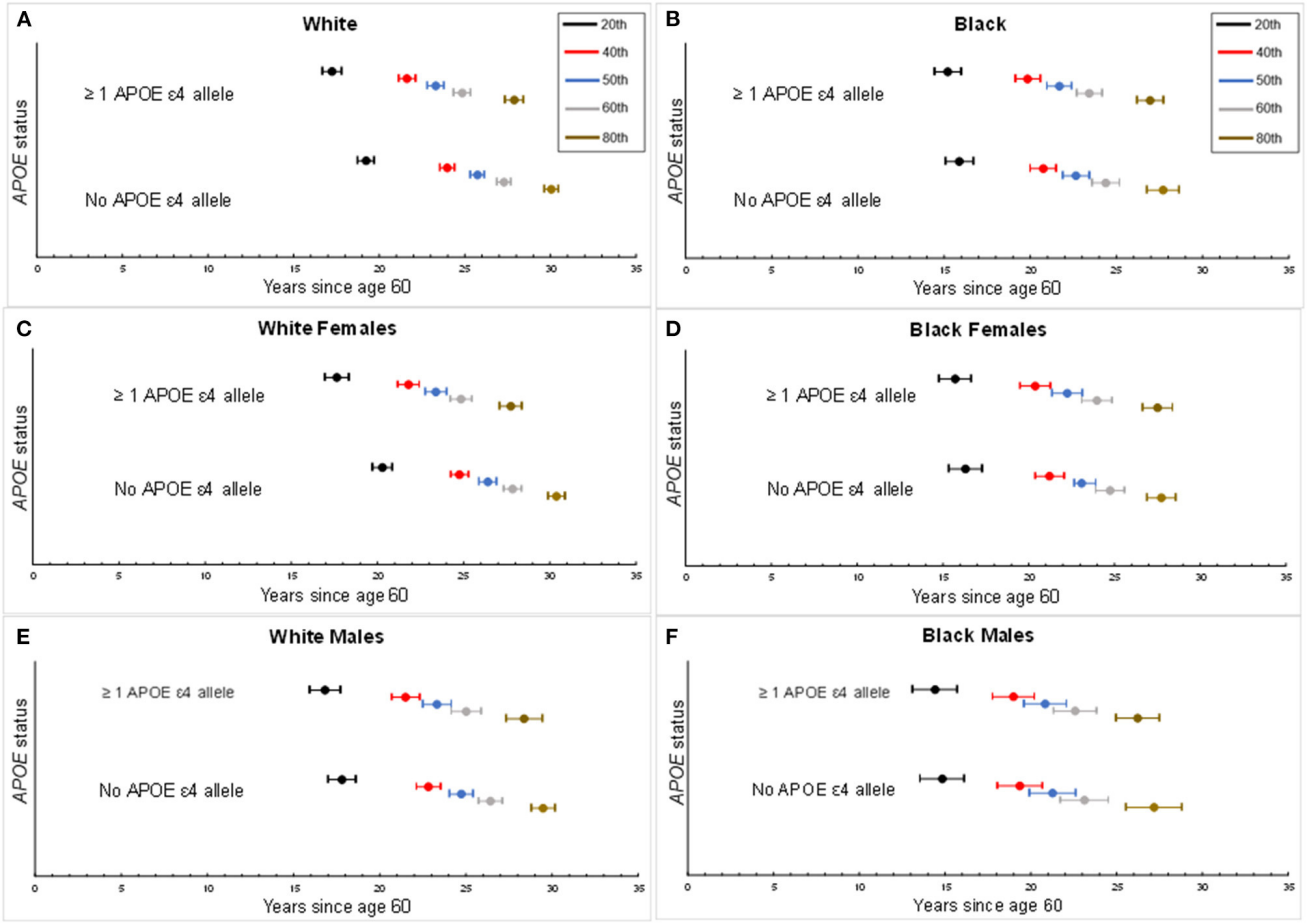
Percentiles for age of dementia diagnosis by *APOE* status. Percentiles of age of dementia diagnosis made by *APOE* ε4 carrier status, accounting for the competing risk of death. Center circle per horizontal bar represents the estimated age of onset of dementia for a given percentile of the study population with the corresponding 95th percentile confidence interval. Each percentile depicts the proportion of dementia diagnosis experienced by age per subgroup. **(A)** Age of dementia diagnosis by White overall study sample. **(B)** Age of dementia diagnosis by Black overall study sample. **(C)** Age of dementia diagnosis for White females. **(D)** Age of dementia diagnosis for Black females. **(E)** Age of dementia diagnosis for White males. **(F)** Age of dementia diagnosis for Black males.

The median age of dementia diagnosis was earlier in ε4 carriers compared with non-carriers, independent of race (Table 2). The median age of diagnosis was 81.7 (95% CI: 81.0, 82.4) for Black carriers or 82.6 years (95% CI: 81.9, 83.4) for Black non-carriers. In Black females, the median age at diagnosis was 82.2 years (95% CI: 81.4, 83.1) on average among carriers and 83.1 years (95% CI: 82.3, 83.9) in non-carriers, whereas in Black males, the median age was 80.8 years (95% CI: 79.6, 82.1) among carriers and 81.3 years (95% CI: 79.9, 82.6) among non-carriers. The median age among Whites was 83.3 years (95% CI: 82.8, 83.8) for White carriers compared with 85.7 years (95% CI: 85.3, 86.1) for White non-carriers. The median age at dementia diagnosis for White females was 83.4 years (95% CI: 82.8, 84.0) in *APOE* ε4 carriers and 86.4 years (95% CI: 85.9, 86.9) in non-carriers. In males, the median age at diagnosis in White carriers was 83.3 years (95% CI: 82.5, 84.2) vs. 84.7 years (95% CI: 84.1, 85.4) in White non-carriers. When considering increasing percentiles of participants with a diagnosis and the age of diagnosis for each subgroup (i.e., a given percentile of dementia diagnosis for a subgroup occurred by the specified age), the age of dementia diagnosis did not differ in Black participants by ε4 allele status (*p*'s > 0.05) but was significantly earlier among White ε4 allele carriers compared with White non-carriers (*p*'s < 0.05; Figure 2). The difference in age at diagnosis by ε4 allele status was greater among older ages, with a greater separation in age at diagnosis over time by ε4 status in White females compared with White males. For both Blacks and Whites, there was no significant difference in age at diagnosis between males and females for ε4 carriers (*p* = 0.9 for Whites; *p* = 0.07 for Blacks). Regardless of race, males experienced an earlier median age of diagnosis of dementia for non-carriers than did females: 81.3 years (95% CI: 79.9, 82.6) vs. 83.1 years (82.3, 83.9) for Blacks and 84.7 years (95% CI: 84.1, 85.4) vs. 86.4 years (85.9, 86.9) for Whites.

**Table 2:** Median age (years) of dementia diagnosis or dementia-free death by *APOE* ε*4 s*tatus in the ARIC cohort (*N* = 13,782)^a^.

	**Dementia**							
	***White*** **(*****n*** **=** **1,778)**				**Black (*****n*** **=** **834)**			
	**≥1** ***APOE*** **ε4 allele**		**No** ***APOE*** **ε4 allele*****s***		**≥1** ***APOE*** **ε4 allele**		**No** ***APOE*** **ε4 alleles**	
	**Age**	**95% CI**	**Age**	**95% CI**	**Age**	**95% CI**	**Age**	**95% CI**
Overall	**83.3**	**(82.8, 83.8)**	**85.7**	**(85.3, 86.1)**	81.7	(81.0, 82.4)	82.6	(81.9, 83.4)
Males	**83.3**	**(82.5, 84.2)**	**84.7**	**(84.1, 85.4)**	80.8	(79.6, 82.1)	81.3	(79.9, 82.6)
Females	**83.4**	**(82.8, 84.0)**	**86.4**	**(85.9, 86.9)**	82.2	(81.4, 83.1)	83.1	(82.3, 83.9)
	**Dementia-free death**							
	**White (*****n*** **=** **3,909)**				**Black (*****n*** **=** **1,571)**			
	**≥1** ***APOE*** **ε4 allele**		**No** ***APOE*** **ε4 alleles**		**≥1** ***APOE*** **ε4 allele**		**No** ***APOE*** **ε4 alleles**	
	**Age**	**95% CI**	**Age**	**95% CI**	**Age**	**95% CI**	**Age**	**95% CI**
Overall	77.9	(77.2, 78.6)	79.9	(79.6, 80.4)	74.1	(73.3, 74.9)	75.8	(74.9, 76.5)
Males	**76.8**	**(76.0, 77.7)**	**79.4**	**(78.9, 79.9)**	73.3	(72.1, 74.5)	74.8	(73.7, 75.8)
Females	79.2	(78.0, 80.3)	80.6	(79.9, 81.3)	**74.8**	**(73.7, 75.9)**	**76.7**	**(75.6, 77.8)**

*CI, confidence interval; ARIC, Atherosclerosis Risk in Communities*.

a *Results account for competing risk of death*.

*Note: Bold indicates difference by APOE status significant at p ≤ 0.05*.

### Risk of Dementia

From competing risk models, compared with *APOE* ε4 non-carriers, the proportion developing dementia (Table 3) was 26% higher among Blacks (46.3% prevalence, 95% CI: 38.3, 54.4%, among carriers vs. 36.6% prevalence, 95% CI: 33.5, 39.9%, among non-carriers). Black females experienced a higher prevalence of dementia than did Black males, regardless of ε4 status, had a higher prevalence in ε4 carriers, with 37.6% (95% CI: 27.1, 49.5%) for male carriers and 52.9% (95% CI: 40.7, 62.5%) for Black female carriers compared with 29.8% (95% CI: 25.9, 34.2%) for male non-carriers and 40.3% (95% CI: 36.6, 44.3%) for Black female non-carriers; however, no significant difference was observed by sex. Among Whites, the overall proportion developing dementia was 38% higher among those with at least one *APOE* ε4 allele (47.0% prevalence, 95% CI: 44.4, 49.6%, among carriers vs. 33.9% prevalence, 95% CI: 32.0, 35.7%, among non-carriers). In White females, 54.5% (95% CI: 50.7, 58.2%) of ε4 carriers developed dementia vs. 41.2% (95% CI: 38.4, 44.0%) of non-carriers (*p* <0.01). Among White males, 40.6% (95% CI: 37.0, 44.3%) and 27% (95% CI: 24.5, 29.1%) of *APOE* ε4 carriers and non-carriers, respectively, developed dementia (*p* < 0.01). While White females had a higher prevalence of dementia diagnosis, there were no significant differences by sex in risk of dementia diagnosis by *APOE* ε4 status.

**Table 3:** Proportion experiencing dementia diagnosis by *APOE* ε*4 s*tatus in the ARIC cohort (*N* = 13,782)^a^.

	**Dementia**							
	***White*** **(*****n*** **=** **1,778)**				**Black (*****n*** **=** **834)**			
	**≥1** ***APOE*** **ε4 allele^b^**		**No** ***APOE*** **ε4 allele*****s***		**≥1** ***APOE*** **ε4 allele^c^**		**No** ***APOE*** **ε4 alleles**	
	**%**	**95% CI**	**%**	**95% CI**		**95% CI**	**%**	**95% CI**
Overall	47.0	(44.4, 49.6)	33.9	(32.0, 35.7)	46.3	(38.3, 54.4)	36.6	(33.5, 39.9)
Males	40.6	(37.0, 44.3)	27.0	(24.5, 29.1)	37.6	(27.1, 49.5)	29.8	(25.9, 34.2)
Females	54.5	(50.7, 58.2)	41.2	(38.4, 44.0)	52.9	(40.7, 62.5)	40.3	(36.6, 44.3)

*CI, confidence interval; ARIC, Atherosclerosis Risk in Communities*.

a *Results account for competing risk of death*.

b *Significantly different by APOE status at p ≤ 0.05 across both sex; no differences noted in overall proportion between sexes for White participants*.

c *Significantly different by APOE status at p ≤ 0.05 across both sex; greater difference noted in proportion of dementia for females compared with males for Black participants*.

### Age at Death

Black *APOE* ε4 carriers experienced a median age of death of 74.1 years (95% CI: 73.3, 74.9) compared with 75.8 years (95% CI: 74.9, 76.5) for non-carriers. Black females had an earlier median age of dementia-free death of 74.8 years (95% CI: 73.7, 75.9) for carriers compared with 76.7 years (95% CI: 75.6, 77.8) for non-carriers (p for difference < 0.01); Black males' median age of death was 73.3 years (95% CI: 72.1, 74.5) for carriers and 74.8 (95% CI: 73.7, 75.8) for non-carriers (p for difference = 0.06).

In comparison, the median age of death for White *APOE* ε4 carriers was 77.9 years (95% CI: 77.2, 78.6) compared with 79.9 years (95% CI: 79.6, 80.4) for White non-carriers. White females on average had a median age of death of 79.2 years (95% CI: 78.0, 80.3) for *APOE* ε4 carriers compared with 80.6 years (95% CI: 79.9, 81.3) for non-carriers (p for difference = 0.04). White males experienced a median age of death of 76.8 years (95% CI: 76.0, 77.7) for *APOE* ε4 carriers compared with 79.4 years (95% CI: 78.9, 79.9) for non-carriers (p for difference < 0.01).

Regarding risk of death by *APOE* status, among those with ≥1 *APOE* ε4 allele, 53.7% (95% CI: 45.6, 61.7%) of Blacks died prior to a dementia diagnosis in carriers, compared with 63.4% (95% CI: 60.1, 66.5%) for non-carriers; 53% of Whites (95% CI: 50.7, 55.6%) with ≥1 *APOE* ε4 allele died prior to a dementia diagnosis compared with 66.1% (95% CI: 64.3, 68.0%) among Whites with no *APOE* ε4 alleles.

### Sensitivity Analysis

When excluding the small number with the ε2/ε2 genotype, a similar risk of dementia diagnosis was observed as that of our overall sample (46.9% White ε4 carriers, 33.8% White non-carriers; 46.3% Black ε4 carriers, 37.2% Black non-carriers). Additionally, the median age of dementia diagnosis was consistent with our overall sample: 83.3 years for White carriers (95% CI: 82.8, 83.8) and 85.8 years for non-carriers (95% CI: 85.3, 86.2) and 81.7 years for Black carriers (95% CI: 80.9, 82.4) and 82.6 years for non-carriers (95% CI: 81.8, 83.4). The estimates of time and risk of dementia by race and sex also remained robust excluding those with the ε2/ε2 genotype.

## Discussion

In this large, population-based cohort of older adults, participants with ≥1 *APOE* ε4 alleles received a dementia diagnosis an average of 1 year earlier for Blacks and 2.5 years earlier for Whites compared with non-*APOE* ε4 carriers. In addition to earlier age of diagnosis, a greater proportion of ε4 carriers experienced a dementia diagnosis compared with non-carriers, confirming the higher risk of incident dementia for ε4 carriers across race and sex. Sex differences were noted in overall risk and timing of dementia diagnosis by ε4 allele status. No significant differences by ε4 carrier status were noted overall in age of dementia diagnosis among Black male or female participants. In contrast, White females experienced a later age of dementia diagnosis (86 years) than did White males (84 years) among non-carriers, but a similar age of diagnosis for ε4 carriers (83 years).

Our findings are consistent with prior work indicating lower cognitive functioning as well as dementia onset and diagnosis at younger ages for carriers of the ε4 allele (23–32), particularly for White individuals. In one of the few additional studies that also accounted for the competing risk of death found in Whites by 85 years of age (11), those heterozygous for ε4 demonstrated an 18.4% greater risk for AD compared with 8.6 or 5.5% for non-carriers depending on ε3 or ε2 status, and even higher risk differences were noted for all-cause dementia.

Our work further acknowledges known racial differences in the effect of the ε4 allele, with a higher prevalence of the ε4 allele in Black individuals but a greater effect on risk of dementia in White individuals (33–35). Additionally, while demonstrating an earlier age of dementia diagnosis in ε4 carriers compared with non-carriers, our results suggest that the average age of dementia onset is not significantly different by sex among Blacks, suggesting that the magnitude of effect of the ε4 allele on age of onset of dementia may be partly race and sex dependent. While no difference in proportion of dementia diagnosis is seen by sex for White individuals, a marginally greater proportion of Black females experienced a dementia diagnosis by ε4 status as compared with Black males. Our results are further consistent with prior research indicating that the greatest driver for age of onset of dementia in Black individuals is not *APOE*, as onset of dementia is earlier in Blacks regardless of ε4 status (34–36).

Work by Rasmussen et al. demonstrated an age-dependent absolute 10-year risk of all-cause dementia for *APOE* ε4/ε4 status at 10 and 8% for 60–69 years and 38 and 33% at 80 years and older (37) among a group of White participants. Females demonstrated a greater overall risk than males; however, a distinction between risk and separate time to dementia was not assessed. In comparison, and in overall agreement with prior work in a biracial cohort (36), our results demonstrated the median age of dementia diagnosis is similar across sex by ε4 status in Whites and indicates a similar earlier shift in age of dementia diagnosis in Blacks, with earlier median age of dementia persisting in Black males. In concordance with our results, Payami et al. (38) demonstrated a shift in age of onset of dementia in female ε4 carriers, but not males. Additional work has suggested (4, 39) that this sex difference in diagnosis rates and age of onset may be explained by the average longer life expectancy in women compared with men. Alternatively, a biological or hormonal basis for differences in risk or onset of dementia has been proposed (15, 40, 41) and may additionally contribute toward the shift in age of diagnosis in White female ε4 carriers seen in our study; however this requires further study in Black participants. Compared with prior work, our analysis allows for investigation of both the onset of dementia diagnosis and assessment of dementia-free death as a competing risk within the same study and among both Black and White participants, further enlightening our understanding of the relationship between *APOE*, dementia, race, and sex.

Many prior studies are primarily clinic-, hospital-, or brain bank-based convenience samples or included participants with family history of dementia—a known risk factor for subsequent dementia. Participants from these settings often have underlying cognitive concerns (42, 43), which can be influenced by genetic risk. In contrast, the ARIC cohort is a community-based sample of adults recruited from age-eligible lists and thus represents a less selected sample of participants with respect to genetic risk and cognitive complaints. The difference in resulting estimates may be attributable to this selection (32). In a meta-analysis across 27 studies, males and females with the ε3/ε4 genotype have overall the same odds of developing MCI or Alzheimer's disease, with some evidence of increased risk for women in certain age ranges. The authors note significant variation in Alzheimer's disease risk between included datasets but remarked that the lowest odds ratios were from community-based studies (44–46) that recruited random participants with no familial relations.

Prior work in a large population-based sample demonstrated that the ε4 allele was associated with increased mortality while the ε2 allele was found to be associated with prolonged survival (47, 48). Our analysis did not consider the homozygous effects of *APOE* ε4 on all-cause dementia, but we would expect the heterozygous effect estimates presented here are conservative in comparison. We conducted a similar analysis of all-cause dementia comparing any ε2 alleles to no ε2 alleles. As expected, results showed a marginal protective effect of the ε2 allele. In the overall sample, Black ε2 carriers experienced a median age of dementia diagnosis 2.2 years later for carriers (median age: 84.1 years carriers, 81.9 years non-carriers) compared with 0.8 years later for White participants (median age: 85.6 years carriers, 84.8 years non-carriers). No significant differences were observed by sex. Both Black and White participants experienced a lower proportion of dementia diagnosis in ε2 carriers, significantly lower for Whites at 32.4 % of carriers, and 38.8% of non-carriers, and marginally lower for Black participants at 37.2% of carriers and 42.0% of non-carriers.

Our results suggest that the ε4 allele has a greater effect than the ε2 on risk of dementia, most notably for White participants. It is worth noting here that race was self-reported by participants. Thus, our distinction of Black vs. White encapsulates more than genetic heritage and additionally includes aspects of culture, geography, socioeconomic status, and health behavior. Given the profound and far-reaching health effects of structural racism, the decreased role of *APOE* ε4 in Black participants may stem from the greater role of social stressors, disparities, and other health risks [e.g., vascular disease (9)] experienced by Black participants in the larger constellation of dementia risk factors relative to White participants. Based on geographic heterogeneity in populations, our results may not be generalizable to all members of racial groups in all areas of the United States.

Known bias in measurement of cognitive function by race exists for some cognitive tests (8). Consequently, it is possible that Blacks may be more likely to be diagnosed with dementia than Whites, particularly for diagnoses made *via* a single cognitive measure. Although it is a strength of the ARIC cohort that surveillance methods are used to ascertain dementia diagnosis for participants who do not return for study visits, it should be acknowledged that some false-positive diagnoses may exist for the participants diagnosed by hospital or death certificate codes, although differences in this bias by race are unclear (33–35).

While the dementia ascertainment protocol incorporated multiple sources of information in ARIC, which allows investigators to assign a dementia diagnosis to all participants and is thus a strength of the study, some cases or dates of diagnosis may be incorrectly identified or be later than actual date of ascertainment. Although it is likely this misclassification error would lead to an overestimation in time to dementia diagnosis and thus underestimation of risk of *APOE* ε4 for dementia, such misclassification would not be expected to differ by *APOE* ε4 status; this means that our results would most likely be biased in a conservative direction (i.e., toward the null).

Our analysis does not account for loss to follow-up of participants who did not return for ARIC study visits and who did not allow for ongoing access to medical records; however, due to the comprehensive nature of dementia and death surveillance within the ARIC cohort, we anticipate that this concern would apply to a small number of participants. Additionally, due to the limited information available for those who were unable to complete neuropsychological testing, we were not able to consider differences in risk of MCI by *APOE* ε4 status. We were unable to investigate etiologic specific subtypes within our data. As our analysis considers incidence of all-cause dementia and not subtypes of dementia, we anticipate that the estimated risk for dementia by genotype will vary by subtype, with likely greater difference in median age of onset or risk for Alzheimer's disease or vascular disease compared with other subtypes due to the known involvement of *APOE* in lipid metabolism (23). Differing mixtures of dementia case types as well as measurement differences in ascertainment of dementia likely contribute to the heterogeneity in prior results and should be targeted areas for future study. Etiologic differences in dementia can have a substantial influence on both onset of disease and proposed management and should be explored in further study.

Death prior to dementia can preclude the observation of dementia, a phenomenon known as a competing risk (12). If an individual dies before a dementia diagnosis, this alters the probability of diagnosis and impedes our ability to observe if they would have received a diagnosis had they not died. By not accounting for competing risks and considering death prior to dementia, the risk estimates for dementia within a given population may be biased (12, 13). We adopted the competing risk method leveraged in this study because of the flexibility of the model and the ability to simultaneously estimate risk of and time to an outcome of interest (i.e., dementia) and another competing outcome (i.e., death). Alternative methods for accounting for competing risk include modeling the cause-specific hazard (49), which provides a description of the event-specific failure among survivors of all possible failures within that population or modeling of the sub-hazard distribution, which provides a description of the cumulative incidences but can easily result in an overestimation of the cumulative incidence (12, 50). While our results are consistent with the sub-hazard model and demonstrated in our sensitivity analysis, the mixture method describes the process as a mixture of conditional distributions by both dementia diagnosis and dementia-free death, thereby providing easier estimation of the time to event and proportion or percentiles with dementia directly, which allows for an estimation of the disease burden. This separation allows for estimation of the proportion (representing risk) and timing of those who were diagnosed with dementia. While not executed for this analysis, the mixture method additionally permits estimation of the cause-specific hazard and sub-hazard distribution, in addition to the relative times and proportions (50). However, the mixture method may not be appropriate for all analyses, as the model requires extensive data to freely estimate the number of parameters required.

Our findings suggest the increased risk is due to both decreased age of diagnosis and proportion experiencing diagnosis but suggests the contribution of each may be modified by both race and sex. These results may be informative for clinical trial planning and recruitment with optimization of timing and age range of participants recruited and informs considerations for recruitment strategies by race and sex, as well as the importance of non-genetic-based risk on dementia risk for Black individuals.

By leveraging a large multiracial population-based cohort with thorough outcome ascertainment and a long duration of follow-up, our findings support prior work showing both elevated risk and shorter time to dementia diagnosis by *APOE* ε4 allele carrier status. We found differences in magnitude of effect of the ε4 allele by both race and sex, with the greatest influence on age of dementia in White females and greatest influence in proportion of dementia cases in Whites overall. This research builds on prior work by using novel statistical methods that allow for the decomposition of the risk and timing estimation of both event types to be interpreted separately, while accounting for the competing risk of death. Our findings further characterize the role of *APOE* in dementia onset and diagnosis as well as the potential influences of sex or race on diagnosis.

## Data Availability Statement

Publicly available datasets were analyzed in this study. This data can be found via application for ARIC Data or through the NIH NHLBI-sponsored Biologic Specimen and Data Repository Information Coordinating Center (BioLINCC) at: https://biolincc.nhlbi.nih.gov/.

## Ethics Statement

The studies involving human participants were reviewed and approved by all participating ARIC Institutions. The patients/participants provided their written informed consent to participate in this study.

## Author Contributions

DP, RQ, SC, MA, JD, and AG contributed to the study concept and design. DP and JD were responsible for the data acquisition. DP, P-LK, RQ, and SC carried out the statistical analyses. DP and P-LK completed the interpretation of the data. DP, JD, RQ, SC, and AG completed the manuscript drafting. DP, P-LK, RQ, SC, DK, PP, RG, MG, JD, and AG were responsible for the manuscript revision. JD and AG contributed to the study supervision. All authors approved the submitted version of the article.

## Funding

The Atherosclerosis Risk in Communities Study was carried out as a collaborative study supported by National Heart, Lung, and Blood Institute contracts (HHSN268201700001I, HHSN268201700002I, HHSN268201700003I, HHSN268201700005I, and HHSN268201700004I). Neurocognitive data are collected by U01 2U01HL096812, 2U01HL096814, 2U01HL096899, 2U01HL096902, and 2U01HL096917 from the NIH (NHLBI, NINDS, NIA, and NIDCD), and with previous brain MRI examinations funded by R01-HL70825 from the NHLBI. This research was support by the National Institute of Health/National Institute on Aging T32AG066576 (DP), the National Institute on Aging Grant K01AG23291 (JD), K01AG050699 (AG), and R00AG052830 (PP).

## Conflict of Interest

The authors declare that the research was conducted in the absence of any commercial or financial relationships that could be construed as a potential conflict of interest.

## Publisher's Note

All claims expressed in this article are solely those of the authors and do not necessarily represent those of their affiliated organizations, or those of the publisher, the editors and the reviewers. Any product that may be evaluated in this article, or claim that may be made by its manufacturer, is not guaranteed or endorsed by the publisher.
